# Immunoreactivity of Sera From Low to Moderate Malaria-Endemic Areas Against *Plasmodium vivax* r*Pvs*48/45 Proteins Produced in *Escherichia coli* and Chinese Hamster Ovary Systems

**DOI:** 10.3389/fimmu.2021.634738

**Published:** 2021-06-24

**Authors:** Myriam Arévalo-Herrera, Kazutoyo Miura, Nora Cespedes, Carlos Echeverry, Eduardo Solano, Angélica Castellanos, Juan Sebastián Ramirez, Adolfo Miranda, Andrey V. Kajava, Carole Long, Giampietro Corradin, Sócrates Herrera

**Affiliations:** ^1^ Immunology Department, Malaria Vaccine and Drug Development Center, Cali, Colombia; ^2^ Immunology Department, Caucaseco Scientific Research Center, Cali, Colombia; ^3^ Laboratory of Malaria and Vector Research, National Institute of Allergy and Infectious Diseases, National Institutes of Health, Rockville, MD, United States; ^4^ Parasitology Department, Centro Nacional de Epidemiología (CNE), Guatemala City, Guatemala; ^5^ Biochemistry Department, University of Lausanne, Epalinges, Switzerland; ^6^ Centre de Recherche en Biologie Cellulaire de Montpellier, Université Montpellier, Montpellier, France

**Keywords:** malaria, *Plasmodium vivax*, gametocytes, transmission blocking, Pvs48/45, vaccines

## Abstract

P48/45 is a conserved gametocyte antigen involved in *Plasmodium* parasite fertilization. A recombinant *Plasmodium vivax* P48/45 (*Pvs*48/45) protein expressed in *Escherichia coli* (*E. coli*) was highly antigenic and immunogenic in experimental animals and elicited specific transmission-blocking (TB) antibodies in a previous pilot study. Here, a similar *Pvs*48/45 gene was expressed in Chinese Hamster Ovary (CHO) cells and we compared its immunoreactivity with the *E. coli* product. Specific antibody titers were determined using plasma from Colombian individuals (n=227) living in endemic areas where both *P. vivax* and *P. falciparum* are prevalent and from Guatemala (n=54) where *P. vivax* is highly prevalent. In Colombia, plasma seroprevalence to CHO-*rPvs*48/45 protein was 46.3%, while for *E. coli*-*rPvs*48/45 protein was 36.1% (*p<*0.001). In Guatemala, the sero prevalence was 24.1% and 14.8% (*p<*0.001), respectively. Reactivity index (RI) against both proteins showed an age-dependent increase. IgG2 was the predominant subclass and the antibody avidity index evaluated by ELISA ranged between 4-6 mol/L. *Ex vivo P. vivax* mosquito direct membrane feeding assays (DMFA) performed in presence of study plasmas, displayed significant parasite transmission-blocking (TB), however, there was no direct correlation between antibody titers and oocysts transmission reduction activity (%TRA). Nevertheless, DMFA with CHO *rPvs*48/45 affinity purified IgG showed a dose response; 90.2% TRA at 100 μg/mL and 71.8% inhibition at 10 μg/mL. In conclusion, the CHO-r*Pvs*48/45 protein was more immunoreactive in most of the malaria endemic places studied, and CHO-r*Pvs*48/45 specific IgG showed functional activity, supporting further testing of the protein vaccine potential.

## Introduction

Malaria is transmitted in ~106 countries in tropical and subtropical regions where over 219 million clinical cases and 405,000 deaths were reported in 2018 (1). Whereas *P. falciparum* is highly prevalent in sub-Saharan Africa, both *P. falciparum* and *P. vivax* coexist in vast regions of Latin America, Asia, and Oceania, with many countries displaying greater *P. vivax* prevalence (2, 3); together these two species are responsible for >90% of the global malaria incidence. In Latin America, 21 countries are malaria-endemic, and in 2018 incidence reached ~632,000 cases; Brazil, Colombia, Peru, and Venezuela accounted for 93% of them (3, 4). Central America experienced a notable malaria reduction (>90%) in the last two decades (2). For example, Guatemala reported 3,521 cases in 2018; most of them (99%) were caused by *P. vivax* (3) and, together with other countries of the region, reached conditions to initiate malaria elimination programs (5). Elimination efforts would greatly benefit from novel and more cost-effective strategies such as vaccines (6), notably transmission-blocking (TB) vaccines (7). It has been observed that individuals continuously exposed to malaria infection in endemic regions develop immune responses that reduce or completely block *Plasmodium* transmission from humans to mosquitoes, which would decrease parasite spreading in endemic populations. This TB immunity is essential to develop vaccines that could accelerate malaria elimination (8, 9).

Several antigens are expressed on the surface of *Plasmodium* gametes (Zygotes/ookinetes) during the fertilization phase in the mosquito midguts (*Pfs/Pvs*25, *Pfs/Pvs28*) (10, 11). Others are expressed in gametocytes when present in the human body (*Pfs/Pvs*48/45, *Pfs/Pvs*230, *Pfs/Pvs*27). These antigens have shown the capacity to elicit specific antibodies capable of blocking parasite transmission to mosquitoes and therefore have been identified as potential TB vaccine candidates (11–13). Among them, *Pfs*25, *Pfs*230, and *Pfs*48/45 are three well-established vaccine candidates tested in preclinical (9, 14, 15) and in phase I clinical trials (16–19).

*Pvs*48/45 is a cysteine-rich conserved protein expressed on the gametocyte surface of several *Plasmodium* species, and is involved in parasite fertilization (20). A full-length version of this protein was formerly expressed in *E. coli* using a codon harmonization approach, and a pilot immunoreactivity study demonstrated that this antigen is recognized by ~60% of the sera from malaria-infected patients, apparently in an age-dependent manner (9). Additionally, immunization studies in BALB/c mice and *Aotus* monkeys indicated high *Pvs*48/45 immunogenicity and elicited antibodies that recognized the parasite protein in immunofluorescence assays and western blotting (WB) analyses. These antibodies also reduce parasite transmission to mosquitoes in *ex-vivo* direct DMFA (14). Despite considerable advantages of the *E. coli* system for recombinant protein production, the accumulation of proteins as inclusion bodies results in the generation of insoluble aggregates of the protein and endotoxin accumulation, among other factors, representing a technical hurdle for expansion of protein production. The CHO cell-line system is commonly used to express large quantities of recombinant proteins (21, 22), and was used here to express the full length *Pvs*48/45 protein. Immunoreactivity of both *E. coli-* and CHO-*rPvs*48/45 proteins was assessed using samples from different malaria-endemic areas of Colombia (COL) and Guatemala (GUA). Additionally, TB activity of anti-CHO-*rPvs*48/45 specific IgG was determined by *ex-vivo* DMFA.

## Material and Methods

### Study Sites

This study was conducted using plasma samples from six sites selected from COL and GUA, previously characterized epidemiologically. Malaria epidemiology of Buenaventura, Tierralta, and Tumaco regions in COL, where both *P. vivax* and *P. falciparum* are endemic (8, 9), and Escuintla, Alta Verapaz, and Zacapa in GUA with almost exclusive (99%) *P. vivax* malaria transmission (23) were studied in a previous NIAID-ICEMR program (8).

### Ethics, Consent, and Permissions

This study was approved by the Institutional Review Board (IRB) at the Centro Internacional de Vacunas (CECIV, Cali-Colombia) in June 2017 (code CECIV 1506-2017) as well as by the Ethics Committee of the GUA Ministry of Health (CNES-dq-005-2015). Additional approval was obtained in GUA from local community leaders before data collection. Written informed consent (IC) was obtained from each volunteer at enrollment. Minors with ages between 7-18 years signed an informed assent (IA) form, and parents or legal guardians gave the corresponding consent. Donors of *P. vivax* infected blood for DMFA were also requested to provide IC.

### Blood Samples

In COL, 227 patients, men (56.8%) and women between 6-84 years of age, were recruited by passive surveillance and included in the study. Subjects were randomly selected among a larger group of patients harboring *P. vivax* or *P. falciparum* infections as determined microscopically and confirmed by qPCR. Whole blood samples (5-15mL) were collected in heparin vacutainer tubes by arm venipuncture, and plasma immediately separated by centrifugation. In GUA, 54 patients with ages between 1-70 years of age (38.9% men) with *P. vivax* infections diagnosed by thick blood smears and confirmed by qPCR, were recruited by active case surveillance, and blood samples were collected as described above. Plasma samples were kept and stored at -80°C until used for serological characterization and for specific CHO-*rPvs*48/45 antibody affinity purification.

### Recombinant *E. coli-* and CHO-*rPvs*48/45 Protein Production

The full-length *rPvs*48/45 gene was expressed in *E. coli*, as described before (24, 25). Briefly, the full codon *Pvs48/45* gene was harmonized using the EuGene software v0.92 (25) and produced by Integrated DNA Technologies (Skokie, IL) into the IDT Blue vector. The synthetic gene was cloned into the pET32a vector for protein expression in Origami 2 (DE3) *E. coli* using a heat shock method (24). The protein of ~60kDa in SDS-PAGE, which corresponds to the predicted weight, was purified by affinity chromatography, using immobilized metal ion affinity chromatography with a histidine cobalt resin (Pierce Inc., USA). Endotoxins were removed using high-capacity removal resin (Pierce, USA). A mass spectrometry (LC-MS/MS) analysis of the protein was performed by using an ion triple quadrupole trap LC-MS/MS with 3200 Q-TRAP (Applied Biosystems, Foster City, CA) (24).

The CHO-*rPvs*48/45 protein was produced by transient gene expression (Excellgene SA, Monthey, Switzerland) with sufficient viable cell culture biomass of suspension adapted CHO-Express™ cells (21). Briefly, the *Pvs48/45* codon harmonized gene was produced in CHO cell lines using the pXLG6 as expression vector, containing a signal peptide at the N-terminus, allowing secretion and a His-6 tag at its C-terminus to allow IMAC purification (or Western detection with an anti-6-His antibody). All production cultures (post-transfection) were performed in serum-free, animal protein-free medium (low protein content). A non-malaria related protein, expressed in parallel during the production of the CHO-*rPvs*48/45 protein, was used as a control production to ensure cell culture and methods accuracy. Each production vessel was verified for critical parameters such as cell culture dynamics and recombinant protein production on samples from day 7 and/or day 14, to confirm the appropriate protein cultures. A total of 150 mg of this antigen was produced after a single purification step on IMAC-FPLC. Two buffer exchanges were performed: one to optimize binding of the protein construct to the IMAC column, and the other one, after elution, to remove excess imidazole. SDS-PAGE analysis of both *E. coli*- and CHO-*rPvs*48/45 proteins under both reducing (0.05 mol/L dithiothreitol) and non-reducing conditions together with immunoblot and mass spectrometry (LC-MS/MS) confirmed the protein identity. Immunoblot analysis was carried out using sera (1:200 dilution) of *P. vivax* semi-immune subjects, plasma from *Aotus* monkeys previously immunized with the *E. coli*-*rPvs*48/45 protein (1:200 dilution), and a E. *coli*-*rPvs*48/45 monoclonal antibody; normal human serum was used as control and LC/MS/MS analysis was performed using peptides generated by trypsin digestion. Peptides were separated and analyzed using a Qexactive-HFX coupled to U3000 RSLC (Thermofisher). Mass spectra were processed with MaxQuant (v1.6.10.43) using a database consisting of Uniprot entries of Cricetulus griseus (UP000001075, 2020_06), 250 classical contaminants and the sequence of the construction.

### ELISA Studies

ELISA was used to determine the two *rPvs*48/45 proteins’ immunoreactivity, as described before (24, 26). Briefly, 96-well plates (Nunc-Immuno Plate, Maxisorp, Roskilde, Denmark) were coated with 1 µg/mL of the two *rPvs*48/45 protein versions in 1x phosphate buffer saline (PBS), pH 7.4 at 4°C overnight. After plates were blocked with milk solution 5%, plasma samples diluted at 1:200 were added and incubated for 1 hour followed by incubation with alkaline phosphatase (1:1000) conjugated goat anti-human IgG antibody (Sigma Chemical Co., St Louis, MO) for 1 hour. Reactions were revealed with para-nitrophenyl phosphate substrate (p-NPP) (Sigma Aldrich) and read at 405 nm wavelength (Dynex Technologies, Inc., MRX Chantilly, VA). Cut-off points for ELISA were calculated as three SD above the mean absorbance value at 405 nm of sera from healthy adult volunteers who had never been exposed to malaria (n=60). The results were also expressed as reactivity index (RI) defined as OD values of tested samples divided by the cut-off value. P-value < 0.05 was considered significant.

### Affinity Purification of Anti-*Pvs*48/45 Antibodies

A pool of selected COL plasma samples known to contain anti-*P. vivax* CHO-*rPvs*48/45 antibodies with ELISA reactive index > 5.0, as described elsewhere (8) was used for purification of anti-CHO-*rPvs*48/45-specific IgG. Briefly, total IgG was purified using a two-step procedure: protein G columns (Pierce, Inc., Rockford, IL), followed by anti-CHO-*rPvs*48/45-specific antibodies purification using an NHS-activated Sepharose column (GE Healthcare Life Sciences, Buckinghamshire, UK) coupled with CHO-*rPvs*48/45 protein, as described previously. Affinity adsorption was performed using the purified total IgG, and elution fraction was neutralized by 1 mol/L Tris, pH 9.0. Specific IgG was then dialyzed against 1x PBS using an Amicon Ultra-4 Centrifugal Filter Unit (30 kDa membrane EMD Millipore). The final protein concentration of specific IgG was measured by DS-11FX+ (DeNovix Inc. Wilmington, DE, USA) and adjusted to 0.5 mg/mL (19). A pool of normal human plasma samples was made from 20 donors, then processed in the same way and used as negative control. Standardized Elisa test was used for quantification of anti-CHO-*rPvs*48/45-specific antibodies (Elisa Units - EU) as described before (27).

### ELISA Competition Assay

Affinity-purified human anti-CHO-*rPvs*48/45 IgG was tested by competition ELISA to determine antibody specificity. Briefly, ELISA microplates were coated with 1 μg/mL of the CHO-*rPvs*48/45 protein and then incubated with the purified antibody; 10 μg/mL of IgG was mixed with increasing concentrations (0 to 100 μg/mL) of the CHO-*rPvs*48/45 recombinant protein competitor. The reaction was incubated for 30 min at RT and the reactivity of the antibody preparation was determined by ELISA as previously described (28). Results were expressed as a percent of binding inhibition of wells without a competitor.

### Avidity Assay

Affinity-purified anti-CHO-*rPvs*48/45 IgG antibodies and four plasma samples from *P. vivax* infected donors (MPV 139: Tierralta, HPV 3031 and P 137: Buenaventura, and P72: Quibdó) displaying ELISA reactivity indices (RI) > 5.0 and four plasma sera from the same regions with RI between 2.0-3.0 (P76, P89, P95, P135) were selected to determine antibody avidity. Polystyrene microplates were coated with 1 μg/mL of the CHO-*rPvs*48/45 protein in duplicates. After overnight incubation plates were washed and blocked with 100 μL of PBS with 0.5% Tween (PBS-T) per well, 5% skim milk (Gibco™, USA) for 2 h at room temperature (RT). Specific IgG at 10 μg/mL or plasma samples diluted at 1:200 in PBS-T 2.5% skim milk was added to the plates and incubated for 2h at RT. After washing, plates were incubated with different urea concentrations (from 0 to 7 mol/L) in duplicates for 15 min to determine antibody avidity. The reaction was revealed using an anti-human IgG alkaline phosphatase conjugate as described above. The urea concentration resulting in 50% of the original ELISA units (IC50) was calculated using linear regression (19).

### IgG Subclass Profile

The *rPvs*48/45-specific IgG subclasses were determined using both CHO and *E. coli* proteins by ELISA as described elsewhere (26). Briefly, 1.0 μg/mL of the corresponding protein was used as antigen to coat microplates, and after blocking with PBS-T plus fat-free milk, plasma sample dilutions were then incubated for 1 hr. Twenty plasma samples were randomly selected from malaria-endemic areas of Colombia, and the purified CHO-*rPvs*48/45-specific human IgG was tested. Plates were washed with PBS-T and then incubated with the following mouse anti-human IgG subclass-specific antibodies: anti-IgG1, IgG3, and IgG4 (Skibio, Bedfordshire, England) and anti-IgG2 (Sigma, St. Louis, MO). Then a peroxidase-conjugated goat anti-mouse IgG antibody (Kierkegaard and Perry Laboratories, Gaithersburg, MD) was added, and the reaction read at 450 nm.

### Direct Membrane-Feeding Assay (DMFA)

The TB activity of 143 plasma samples (1:2 dilution), and affinity-purified CHO-*rPvs*48/45 specific IgG antibodies at 10, 40, and 100 μg/mL was measured by a DMFA using *P. vivax* gametocyte-infected blood obtained from malaria patients, as previously described (9). Briefly, *P. vivax* infected blood was centrifuged, and cell-pellets containing infected RBC (iRBC) were subsequently reconstituted with a pool of heat-inactivated male AB+ sera obtained from healthy donors purchased from Sigma (H5667 Sigma-Aldrich Inc) or with test samples. A total of ~100 *Anopheles albimanus* mosquitoes previously subjected to overnight fasting were fed for 15-20 minutes/cage with this mixture. Mosquito midguts were stained with 2% mercurochrome on day 7 post-feeding, and the number of oocysts per mosquito midgut counted.

### Statistical Analysis

All statistical tests were performed in Prism 6 (GraphPad Software), and *p*-values <0.05 were considered significant. Antibody responses (seroprevalence and percentage of RI>2) for both CHO-*rPvs*48/45 and *E. coli-* were compared by Fisher’s exact test. Differences of RI between age groups were analyzed by Kruskal-Wallis, followed by Dunn’s multiple comparison test. Correlation between %TRA of human sera and RI by Spearman test. Avidity (IC50) of *P. vivax* plasma samples and affinity-purified CHO-*rPvs*48/45 specific IgG were compared by Mann-Whitney tests (20).

The percentage reduction in mean oocyst count/mosquitoes (TRA) was calculated using the following formula: [(Xc − Xa)/Xc)] × 100, where X is the arithmetic mean oocysts in control (c) and test (a) plasma or IgG. The 95% confidence interval and *p*-value for TRA was calculated using a zero-inflated negative binomial model as previously described (29).

## Results

### Expression and Purification of the *rPvs* 48/45 Proteins

**Figure 1:**
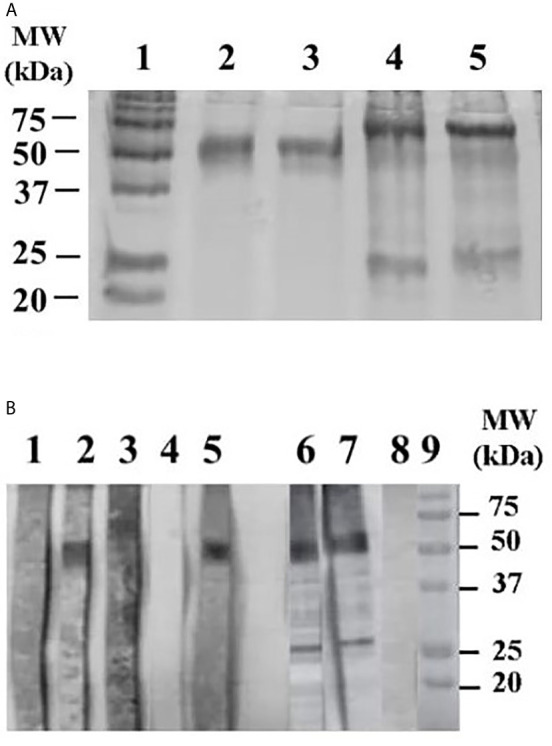
**(A)** 12% SDS-PAGE gel of CHO- and *E. coli-rPvs*48/45 proteins stained with Coomassie blue. Lane 1, molecular weight markers. Lane 2 and 3, CHO-*rPvs*48/45 protein (~ 50 kDa) under non-reducing and reducing conditions (0.05 M dithiothreitol) respectively. Line 4 and 5, *E. coli*-*rPvs*48/45 proteins (~ 60 kDa) under non-reducing and reducing conditions, respectively. **(B)** Western blot analysis under non-reducing conditions of the CHO-*rPvs*48/45 protein against PBS solution. Lane 1, Normal human sera, lane 2 sera from *P. vivax* malaria immune subject, lane 3, Normal monkey plasma, lane 4, Blank, lane 5, monkey plasma immunized with *E coli-rPvs*48/45 from a previous study (25). Lane 6, 7 and 8 western blot analysis under non-reducing conditions of the *E. coli-rPvs*48/45 protein using a mouse monoclonal, a primate polyclonal anti-*rPvs*48/45 antibody and Normal monkey plasma respectively. Lane 9, molecular weight markers.

Amplification of the *E. coli*-*rPvs*48/45 gene without the signal peptide and GPI anchor was sub-cloned in the *E. coli* system as described elsewhere (24). Both the affinity-purified CHO- and the *E. coli*-*rPvs*48/45 protein products were analyzed by SDS-PAGE under non-reducing conditions, indicating the presence of a ~50kDa and ~60kDa bands, respectively, corresponding to the expected product (**Figure 1A**) as previously described for P48/45 proteins (24, 30). The different MW of the two proteins is due to: 1) an additional thioredoxin protein fragment added to the *E. coli* protein (108 aa) inserted to avoid inclusion bodies formation (24, 31) whereas the CHO-*rPvs*48/45 has a shorter thioredoxin fragment (42aa), and a theoretical mass of 51,766.22 (see **Supplementary Material**). Both proteins have a ~89% similar sequence and were both recognized by sera from *P. vivax* malaria semi-immune subjects as well as from plasma of *Aotus* monkeys immunized with the *E. coli*-*rPvs*48/45 protein (**Figure 1B**: lane 1 and 2), and confirm the similarity of both proteins and previous results (24). In addition, a lower molecular band at the 25kDa appeared in the *E. coli* product in both reduced and non-reducing conditions and the western blot analysis under non-reducing conditions of this protein showed the recognition of this lower band by both a mouse monoclonal and a primate polyclonal anti-*rPvs*48/45 antibody (**Figure 1B**: lanes 3 and 4).

### Immunoreactivity

**Figure 2:**
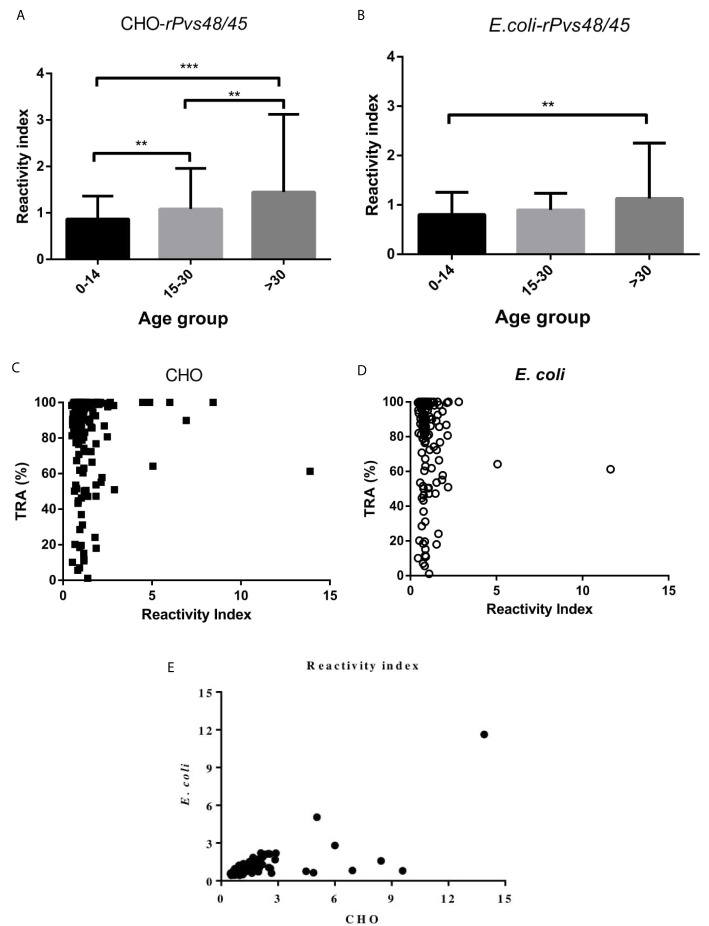
Age-dependent reactivity index **(A, B)**, and correlation between percentage inhibition in oocyst density (%TRA) of human sera and reactivity index of COL and GUA **(C, D)** to *rPvs*48/45 expressed in both CHO ***(*A, C)** and *E. coli* **(B, D)**. **(A, B)** the bars and error bars correspond to mean values and 2SD. Statistical significance was observed between each age group in both proteins (*p* < 0.0001) by Kruskal Wallis analysis followed by Dunn’s multiple comparison tests between two groups. Bars indicated significant difference between groups of 0-14 and >30 years of age (***p < 0.0001; **p < 0.05). **(C, D)** show no correlation between %TRA and RI (CHO protein *p*>0.28 and *E. coli p* > 0.63) by Spearman tests. **(E)** A dot blotting chart showing the reactivity index of each sera for both proteins.

From the total plasma samples studied (n= 281), ELISA assays indicated the presence of specific antibodies to the full-length CHO-*rPvs*48/45 in 42% (n=118) of samples and in 32% (n=90) to the *E. coli*-*rPvs48/45* product (*p*<0.001) (**Table 1**). Immunoreactivity against both proteins was higher in the COL than in the GUA samples (CHO, *p*=0.0030, and *E. coli*, *p*=0.0026). In COL plasmas (n=227) the prevalence of specific antibodies was 46.3% and 36.1% to the CHO and *E. coli* products, respectively. In contrast, in GUA plasmas (n=54) it was 24.1% and 14.8%, respectively, indicating significant regional differences for both proteins (CHO and *E. coli p*<0.001). Also, samples from Tierralta, (COL), where *P. vivax* is highly prevalent (2,630 cases/100,000 habitants; 65% *P. vivax)*, presented an immunoreactivity of 58.8% and 34.3% against the CHO and *E. coli* proteins, respectively. In contrast, samples from Tumaco, where *P. falciparum* is more prevalent (1,338 cases/100,000 habitants; 96.2%) than *P. vivax* (INS, 2018), ELISA showed 27.3% and 41.7%, respectively. Significant differences in prevalence among both proteins in samples from COL (Tierralta: *p*<0.001, Buenaventura: *p*<0.002) and GUA samples (*p*<0.001) was observed. In contrast, in Tumaco (COL) *E. coli* was better recognized than CHO (*p*<0.001) (**Table 1**). In Colombia, the seroprevalence of samples with RI > 2.0 was found in 9.7% (n=22) of the samples tested with the CHO protein, and 4.0% (n=9) in those tested with the *E. coli* protein (*p*<0.001). In GUA, people with > 2.0 RI were 8% (n=1) and 0% (n=0) to CHO and *E. coli* protein, respectively. Fisher tests indicated that plasma samples with RI > 2 were significantly more frequent in Colombia than in GUA (*p*=0.0042) to CHO protein. In contrast, there was no difference between both countries to *E. coli* protein. To determine the impact of age on the RI, in both COL and GUA, samples were stratified into three groups: 0-14 years/old n=41 (14.6%); 15-30 year/old n=118 (42%); and more than >30 years/old, n=122 (43.4%). The mean RI presented an age-dependent increasing trend (**Figures 2A, B**), which was more significant when plasma samples were tested using the CHO-*rPvs*48/45 protein (*p*<0.05). Individuals >30 years/old presented a RI to CHO mean of 1.45 whereas to *E. coli*, the mean was 1.13. Significant differences (*p*<0.05) were observed in the RI values between children (<15 years/old) and adults (>30) when the *E. coli* - *rPvs*48/45 protein was used (CHO = 0.86; *E. coli* = 0.81). Regarding TRA activity, 93 of the 143 (65.03%) plasma samples examined by DMFA displayed >80% TRA; 51samples (54.83%) were reactive to the CHO- and 29 (31.18%) reactive to *E. coli-rPvs*48/45 protein (**Figure 2E**). As shown in **Figures 2C, D** no correlation was found between % TRA activity and RI values in both proteins (Spearman test; CHO protein *p*>0.28 and *E. coli p*>0.63).

**Table 1:** Prevalence of antibodies reactive to CHO-*rPvs*48/45 and *E. coli* in plasma samples of subjects from Colombia and Guatemala.

		CHO	*E. coli*	*p*-value^c^	CHO	*E. coli*	*p*-value^c^
		Seroprevalence n (%)^a^	Seroprevalence n (%)^a^		RI >2 n (%)^b^	RI >2 n (%)^b^	
**Site**	**n**						
**COLOMBIA**							
Tierralta	102	60 (58.8)	35 (34.3)	<0.001	14 (13.7)	4 (3.9)	<0.001
B/ventura	41	22 (53.7)	12 (29.3)	0.002	7 (17.0)	4 (9.8)	<0.001
							
Tumaco	84	23 (27.3)	35 (41.7)	<0.001	1 (1.2)	1 (1.2)	0.012
**Sub-total**	**227**	**105 (46.3)**	**82 (36.1)**	<0.001	**22 (9.7)**	**9 (4.0)**	<0.001
**GUATEMALA**	54	13 (24.1)	8 (14.8)	<0.001	1 (1.8)	0 (0.0)	
**Total samples**	**281**	**118 (42.0)**	**90 (32.0)**	**<0.001**	**23 (8.2)**	**9 (3.2)**	**<0.001**

^a^Correspond to number and percentage of malaria exposed subjects. Percentage of positive responses: OD values above the negative control mean plus 3SD. Negative control: with no malaria history.

^b^Seroprevalence of reactivity index (RI > 2) between the mean of the experimental and the mean of control plasma OD. ^c^Fisher’s exact test calculated p-value (significant p < 0.05) between seroprevalence of all the plasma samples and seroprevalence of plasma samples with RI>2 in both rPvs48/45 protein (CHO and E. coli). In bold: E. coli, Escherichia coli; CHO,Chinese hamster ovary.

### The Specificity of Affinity-Purified Anti-CHO-*rPvs*48/45-Specific Antibodies

**Figure 3:**
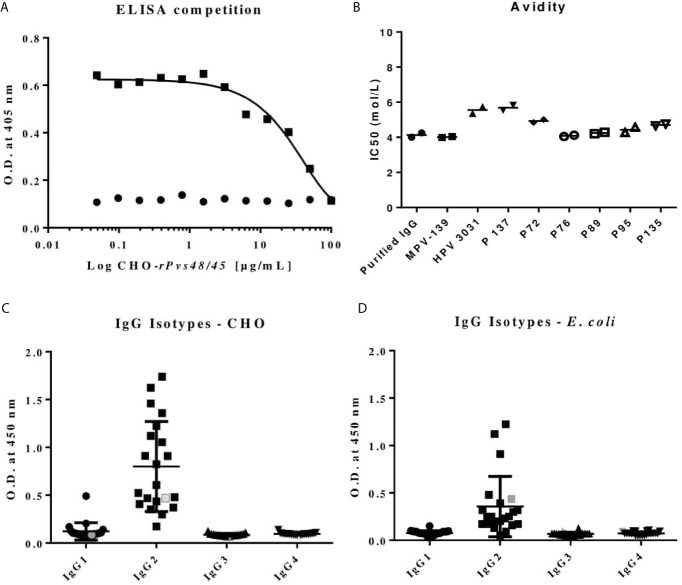
**(A)** ELISA competition assay for the affinity-purified CHO-*rPvs*48/45-specific human IgG (▪) and normal IgG (•) against CHO-*rPvs*48/45 protein. Plates were coated with CHO-*rPvs*48/45 recombinant protein (1 μg/mL) and then a test IgG (10 μg/mL) was incubated together with the recombinant protein at different concentrations (0 to 100 μg/mL). **(B)** The IC50 mean of purified IgG and sera samples were determined by ELISA with increasing urea concentration (from 0 to 7 mol/L). The serum samples were selected for presenting the highest reactivity index (RI>5) of the sampling location: MPV 139 (Tierra Alta), HPV 3031 and P 137 (Buenaventura), and P72 (Quibdó) and reactivity index between 2-4: P76, P89, P95, P135 from the same regions. Anti-IgG1, IgG2 IgG3, and IgG4 subclasses are reported as OD mean of COL plasma samples (black symbols) randomly chosen and purified CHO-*rPvs*48/45-specific human IgG (grey symbols) using plates coated with either CHO- **(C)** or *E. coli-rPvs*48/45 **(D)** protein.

To investigate characteristics and functionality of the protein, specific antibodies were affinity purified from a pool of plasmas with high RI (>5.0) using the CHO r*Pvs*48/45 protein. A total of 40 mg/mL of total IgG was obtained after protein G column purification and 500ug/mL of anti-CHO-*rPvs*48/45-specific antibodies after Sepharose column purification with specific antibody titers of 62.163 EU (27). Specificity of anti-CHO-*rPvs*48/45 specific antibodies was confirmed by ELISA competitive assay as shown in **Figure 3A**. At 50 μg/mL, the CHO r*Pvs*48/45 competitor protein induced a remarkable inhibition when the purified specific-IgG was used at 10 μg/mL. i.e., ~80% of inhibition. In the IgG subclass analysis, IgG2 was the most abundant, both in affinity-purified anti-CHO-*rPvs*48/45 antibody preparation, as well in the plasma of donors with positive antibody titers to both CHO- (n=21) (**Figure 3C**) and *E. coli-rPvs*48/45 proteins (n=22) (**Figure 3D**).

To determine the protein avidity, anti-CHO- and *E. coli*-*rPvs*48/45 specific IgG and sera samples from malaria-endemic regions were incubated with different concentrations of urea. All data sets fitted into the linear regression model (R^2^>0.94), and the anti-*rPvs*48/45 specific IgG presented a similar IC50 (4.1 and 4.5 mol/L respectively) with plasma samples with RI between 2-4 (P-76: 4.1 mol/L, P-89: 4.3 mol/L, P-95: 4.4 mol/L) and a slightly higher avidity with the HPV-3031 (5.5 mol/L), P-137 (5.6 mol/L), and P-72 (4.9 mol/L) of plasma samples with RI>5.0, except with the sample MPV-139 (3.9 mol/L) (**Figure 3B**).

### Transmission-Blocking Activity

Two independent DMFA assays were performed to determine oocyst reduction by specific IgG. CHO-*rPvs*48/45-specific IgG showed significant reduction in the mean oocyst numbers, compared to normal human IgG; and the inhibition was dose-dependent (**Table 2**). TRA increased from 71.8% to 90.2% when the IgG concentration was increased from 10 to 100 μg/mL.

**Table 2:** DMFA with human anti-CHO-*rPvs*48/45-specific IgG^a^.

IgG conc. [µg/mL]	mean oocyst number in Assay 1		mean oocyst number in Assay 2		^b^best estimates of %TRA from two assays		
	Normal IgG	*rPvs48/45* IgG	Normal IgG	*rPvs48/45* IgG	%TRA	(95%CI)	p-value
10	9.1	1.9	7.0	2.6	71.8	(51.6 - 84.0)	<0.001
40	9.8	1.6	5.9	2.5	74.0	(55.0 - 85.1)	<0.001
100	9.7	0.4	4.8	1.3	90.2	(82.4 - 95.4)	<0.001

a Human normal and anti-CHO-rPvs48/45-specific IgGs were tested at indicated concentrations in two independent assays. Forty mosquitoes were examined to determine the mean oocyst density of a group.

b The best estimate of %TRA, the 95% confidence interval (95%CI), and p-value for the anti-CHO-rPvs48/45 IgG at each concentration were calculated using a zero-inflated negative binomial model as described previously (28).

## Discussion

This study confirms that plasma of a significant proportion of individuals living in malaria endemic areas of COL and GUA recognize the *Pvs*48/45, independently of the recombinant product used as antigen in the ELISA and about half of these plasma samples had the capacity to *ex vivo* block *plasmodium* transmission to *Anopheles* mosquitoes.

The results here where both *P. vivax* and *P. falciparum* coexist in most endemic regions are consistent with those obtained with the *P. falciparum* orthologous protein (*Pfs*48/45) in studies conducted in several countries in Africa where this species is almost exclusively transmitted and malaria transmission intensity is significantly higher than in Latin America (29, 32–34). In this study, we included GUA, where malaria is virtually produced exclusively by *P. vivax*, and COL where the study sites have transmission of both *P. vivax* and *P. falciparum*, in different proportions (35, 36). Likewise, it confirms previous results on the immunoreactivity of the *rPvs*48/45 expressed in *E. coli* and the *ex vivo* TB activity studied of plasma samples from endemic areas of COL (24) and from Africa (37).

The frequent presence of antibodies to r*Pvs*48/45 in the study sites, known to be regions of low to intermediate malaria transmission intensity, is encouraging as it indirectly reflects the protein’s immunoreactivity in natural conditions, even when parasite exposure is low or moderate. Nevertheless, we found that the protein recognition is associated with the malaria transmission intensity in the study areas. i.e., it was higher in COL (106 cases/100,000 habitants) than in GUA (22 cases/100,000 habitants) in the year 2017 (PAHO, 2020). In this regard, in COL, immunoreactivity was higher and more prevalent in areas with the relatively higher transmission, e.g., in Tierralta (2,630 cases/100,000 habitants), Department of Cordoba (38). However, it is intriguing that in GUA that has reduced the malaria incidence by ~95% since 2000 (5, 23), individuals from endemic regions still present significant immunoreactivity with r*Pvs*48/45. Colombia presented an incidence reduction of ~50% between 2000 and 2015 but still reports >60,000 clinical cases/year with >60% of the cases reported in the study areas, the Pacific coastal region and Cordoba department (39). This is coherent with the age-dependent trend of specific antibodies; subjects >30 years of age showed the highest RI against the *rPvs*48/45 protein.

Although both proteins were highly immunoreactive, CHO was significantly better recognized by all age groups, except in Tumaco (COL) where *E. coli* was significantly better recognized. The different immunoreactivity of the two proteins might be explained by 1) the conformational differences between the 3D structures of the two proteins and, thus, recognition of different epitopes, 2) by the additional thioredoxin fragment added to the *E. coli* protein to improve its expression, 3) by potential glycosylation in the CHO protein.

None of the constructs were recognized by sera from naïve individuals indicating that neither full-length *Pvs*48/45and nor the additional fragment are recognized by control sera.

Another intriguing observation is that in Tumaco (COL) the prevalence of *P. falciparum* is significantly higher that *P. vivax*, yet, the response to *rPvs*48/45 was similar than in Tierralta (COL) and GUA suggesting a parasite cross-reactivity between the orthologous proteins in natural conditions. To better understand the sero-epidemiology of the *rPvs*48/45, we are currently studying malaria-endemic regions with different *P. vivax/P. falciparum* proportions.

Although TB activity displayed by whole plasma can be the result of antibodies to multiple parasite antigens (**Figures 2C, D**), here, strong inhibition was induced by affinity purified anti-CHO-*rPvs*48/45 antibodies which confirmed the important role of this parasite antigen in TB immunity under natural conditions (8, 9). It is also remarkable that the DMFA performed with the affinity-purified antibodies displayed TRA dose responses (**Table 2**). This is an indirect indication, first, of the correct conformation of the protein and second, that anti-*rPvs*48/45 antibodies induced by natural infection retained the functional activity after purification.

The two proteins displayed different profile at the SDS-PAGE gel by the presence of two bands of high (~50kDa) and a low (~25kDa) molecular weight in the *E. coli* product. Previous studies using both recombinant and natural proteins have observed the same pattern (40, 41). Here it was confirmed that the smaller fragment corresponds to a proteolytic product of the protein by the blot reactivity with both a monoclonal and a polyclonal anti-*Pvs*48/45 antibody. Therefore, as this protein develops as vaccine candidate purification process must be optimized.

Although several IgG subclasses may contribute to the overall TB activity, the high prevalence of IgG2 suggests that anti-*rPvs*48/45 activity is independent of the classical complement; this issue requires further analyses.

The results described here, together with the progress achieved with the *Pfs*48/45 vaccine candidate (42, 43) and previous results of sera from mice and monkeys previously immunized with the *rPvs*48/45 *E. coli* version encourage further studies on the vaccine potential of this *P. vivax* protein. It is of great importance to conduct studies using plasma from areas where *P. falciparum* is exclusively transmitted, i.e., Africa, which may contribute to elucidate the potential cross-reactivity of the protein in the field. If the cross reactivity in nature were accompanied by cross functional activity, i.e. TB activity, it would be of great importance for vaccine development.

## Data Availability Statement

The raw data supporting the conclusions of this article will be made available by the authors, without undue reservation.

## Ethics Statement

The studies involving human participants were reviewed and approved by Institutional Review Board (IRB) at the Centro Internacional de Vacunas (CECIV, Cali-Colombia) (code CECIV 1506-2017) Ethics Committee of the GUA Ministry of Health (CNES-dq-005-2015). Written informed consent to participate in this study was provided by the participants’ legal guardian/next of kin.

## Author Contributions 

Conceived and designed the experiments: MA-H and SH. Performed the experiments: NC, CE, KM, ES, AC, JR, and AM. Analyzed the data: MA-H, SH, GC, and CL. LC/MS/MS CHO-*rPvs48/45* analysis: AK and GC. Contributed reagents/materials/analysis tools: MA-H, NC, and SH. Wrote the paper: MA-H, NC, GC, CL, and AK. All authors contributed to the article and approved the submitted version.

## Funding

NIH/NIAID 1R01AI121237-01 sponsored this study and in part by the Intramural Research Program of NIAID, NIH.

## Supplementary Material

The Supplementary Material for this article can be found online at: https://www.frontiersin.org/articles/10.3389/fimmu.2021.634738/full#supplementary-material

## Conflict of Interest

The authors declare that the research was conducted in the absence of any commercial or financial relationships that could be construed as a potential conflict of interest.
