# Expression of concern on: Immunoreactivity of sera from low to moderate malaria-endemic areas against plasmodium vivax rPvs48/45 proteins produced in *Escherichia coli* and Chinese hamster ovary systems

**DOI:** 10.3389/fimmu.2023.1292349

**Published:** 2023-09-15

**Authors:** Frontiers Editorial Office

With this notice, Frontiers states its awareness of serious allegations surrounding the institutional review board Centro Internacional de Vacunas cited in this article. These allegations are being investigated in line with COPE guidelines. The situation will be updated as soon as the investigation is complete.

