# Supplementary figures and images for: Immunoreactivity of Sera From Low to Moderate Malaria-Endemic Areas Against Plasmodium vivax rPvs48/45 Proteins Produced in Escherichia coli and Chinese Hamster Ovary Systems
Source: Front Immunol. 2021 Jun 24;12:634738. doi: 10.3389/fimmu.2021.634738 (PMC8264144; doi:10.3389/fimmu.2021.634738)

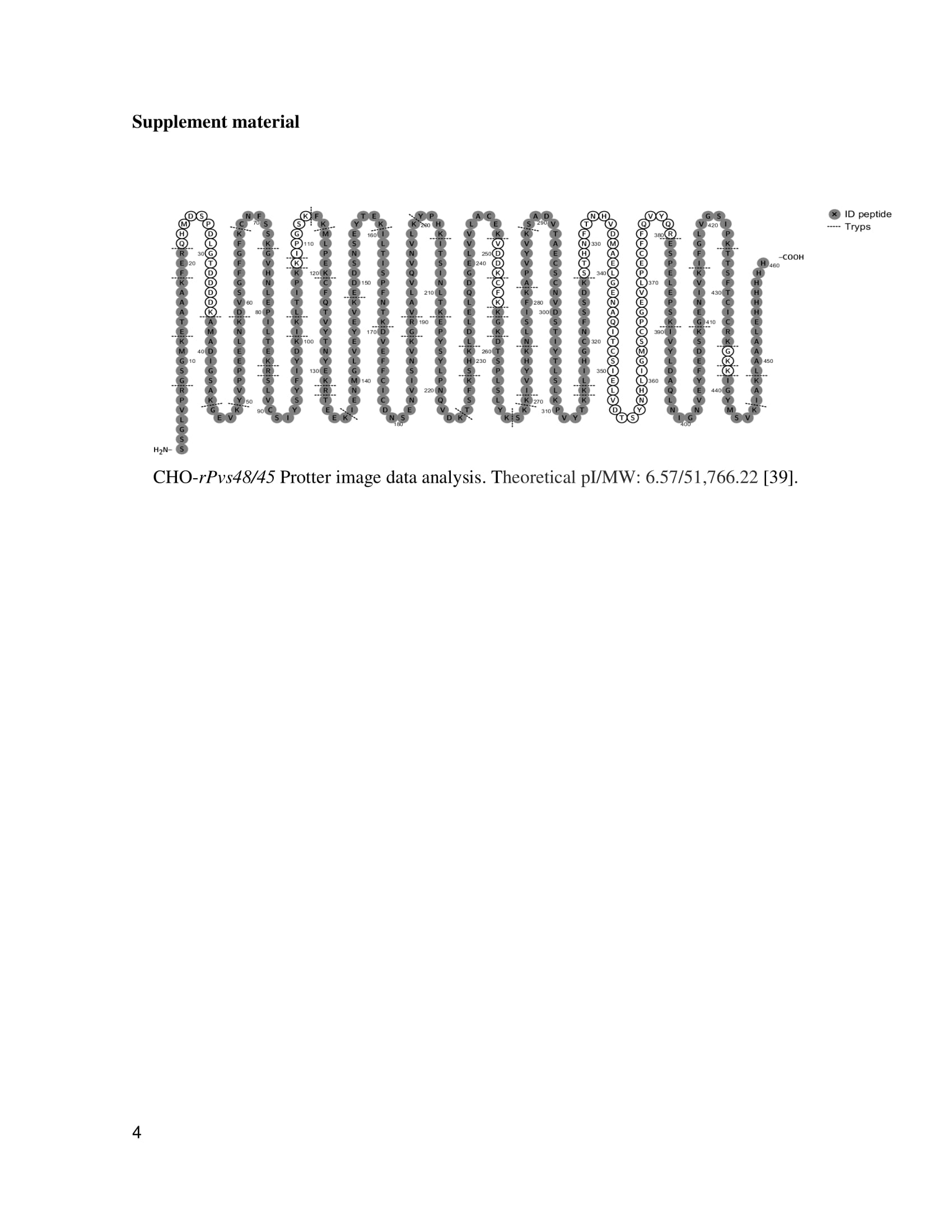

Supplement: Supplementary file 1 [file Image_1.jpg]
